# Large-scale application of palaeoproteomics (Zooarchaeology by Mass Spectrometry; ZooMS) in two Palaeolithic faunal assemblages from China

**DOI:** 10.1098/rspb.2023.1129

**Published:** 2023-10-25

**Authors:** Naihui Wang, Yang Xu, Zhuowei Tang, Cunding He, Xin Hu, Yinqiu Cui, Katerina Douka

**Affiliations:** ^1^ School of Life Sciences, Jilin University, 130012 Changchun, People's Republic of China; ^2^ School of Archaeology, Jilin University, 130012 Changchun, People's Republic of China; ^3^ Max Planck Institute of Geoanthropology, 07745, Jena, Germany; ^4^ Department of Early Prehistory and Quaternary Ecology, University of Tübingen, Schloss Hohentübingen, 72070 Tübingen, Germany; ^5^ China-Central Asia ‘the Belt and Road’ Joint Laboratory on Human and Environment Research, 710127 Xi'an, People's Republic of China; ^6^ School of Cultural Heritage, Northwest University, 710127 Xi'an, People's Republic of China; ^7^ Chongqing China Three Gorges Museum, 400013 Chongqing, People's Republic of China; ^8^ Department of Evolutionary Anthropology, Faculty of Life Sciences, University of Vienna, 1030 Vienna, Austria; ^9^ Human Evolution and Archaeological Sciences (HEAS), University of Vienna, 1030 Vienna, Austria

**Keywords:** ZooMS, palaeoproteomics, deamidation, radiocarbon dating, Palaeolithic, camels

## Abstract

The application of Zooarchaeology by Mass Spectrometry (ZooMS) on Pleistocene sites in Europe and northern Asia has resulted in the discovery of important new hominin fossils and has expanded the range of identified fauna. However, no systematic, large-scale application of ZooMS on Palaeolithic sites in East Asia has been attempted thus far. Here, we analyse 866 morphologically non-diagnostic bones from Jinsitai Cave in northeast China and Yumidong Cave in South China, from archaeological horizons dating to 150–10 ka BP. Bones from both sites revealed a high degree of collagen preservation and potentially time-related deamidation patterns, despite being located in very distinct environmental settings. At Jinsitai, we identified 31 camel bones, five of which were radiocarbon dated to 37–20 ka BP. All dated specimens correspond to colder periods of Marine Isotope Stages 3 and 2. We regard the presence of camels at Jinsitai as evidence of wild camels being a megafauna taxon targeted, most likely by early modern humans, during their expansion across northeast Asia. This large-scale application of ZooMS in China highlights the potential of the method for furthering our knowledge of the palaeoanthropological and zooarchaeological records of East Asia.

## 1. Introduction

Significant new archaeological and palaeoanthropological discoveries from East Asia have highlighted the region's importance in understanding late human evolution [1–5]. However, our knowledge of human presence and adaptation to these, often extreme, territories are limited, although some multi-period sites with long stratigraphies offer such potential (e.g. [6]).

Recent developments in ancient DNA (aDNA) research including the extraction of aDNA from sedimentary deposits and bone remains, have opened new exciting possibilities worldwide [7,8]. However, aDNA preservation is challenging in some regions of East Asia, particularly in warm and humid areas. Ancient proteins are an alternative group of biomolecules that often preserve better and can help address research questions in palaeoanthropology and zooarchaeology [9,10]. Peptide mass fingerprinting of collagen, also known as zooarchaeology by mass spectrometry (ZooMS), is a powerful palaeoproteomic method for the taxonomic identification of collagenous materials such as bone, ivory and leather [11–14]. ZooMS involves the extraction of Type I collagen (COL1) and the generation of tryptic-digested peptide mass fingerprints using matrix-assisted laser desorption ionization time-of-flight mass spectrometry (MALDI-TOF-MS). COL1, the major organic component (approx. 90%) in the bone of vertebrates, is a highly durable biomolecule, and peptides as old as 3.5 Myr have been extracted from bone remains [15].

ZooMS is particularly valuable analytical tool for screening highly fragmented bones that lack diagnostic features and therefore are not suitable for traditional zooarchaeological analyses [16–21]. The method performs well on bones from cold environments, while its success rates for bones from temperate, tropical and subtropical zones are generally lower [22].

In this work, we investigate the applicability of ZooMS in East Asia as part of a larger-scale, study involving numerous Pleistocene-age sites from across Eurasia (FINDER Project). The aims of our work in China were threefold. First, we wanted to examine whether the application of ZooMS on various Chinese sites—where the method had not been applied on a large scale before—would be successful and whether site location and age would be a major contributing factor to success or failure rates. Assuming a degree of successful collagen extraction, the second aim of this project revolved around the identification of new hominin remains and, finally, the third aim was an attempt to expand the morphology-based faunal identifications using ZooMS. While our initial goal was to include a large number of sites and bone material from different periods and depositional environments, the pandemic prevented us from studying a larger number of sites. Despite this limitation, this work, designed as a feasibility study for the recovery of ancient proteins from different locations in China, represents the largest application of ZooMS in East Asia to date.

## 2. Material and methods

**Figure 1.**
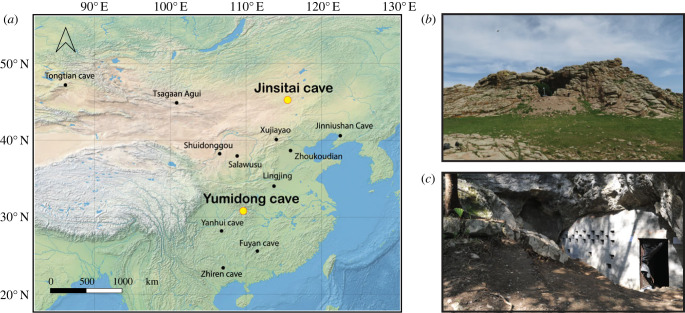
Map with location of sampled sites in this study. (*a*) Location of Yumidong Cave and Jinsitai Cave shown with yellow dots; (*b*) view towards Jinsitai Cave; (*c*) the entrance of Yumidong Cave. Base map from https://www.naturalearthdata.com/.

We applied ZooMS to 866 unidentified bones from two Palaeolithic sites in China: Yumidong Cave in the south and Jinsitai Cave in the north (figure 1). Information about the sites, the analysed material and methods of analyses are detailed below.

### (a) Studied sites

The two analysed sites are located in regions with distinct ecological and climatic settings, (assumed) biomolecular preservation conditions and research histories.

The Three Gorges region is a hub for archaeological and paleoanthropological research, with many sites having been discovered in recent decades. Yumidong Cave is a recently found karst cave in this region [23]. It consists of a large and nearly horizontal chamber, 70 m in length and 12–20 m in width. A 3 m in diameter vertical skylight provides air circulation and light, making the cave particularly attractive to human occupation. Excavations began in 2011 and focused on the area between the roof skylight and the cave entrance. Approximately 150 m² of surface area has been exposed thus far. The stratigraphy consists of a 6 m depth sequence divided in 18 distinct layers; the bedrock has not been reached (electronic supplementary material, appendix and figure S1). The excavations have yielded thousands of lithics and fauna remains, including 113 worked bones. Large limestone tools make up 97% of the lithic assemblage and belong to the cobble industry that prevailed in southern China during the Pleistocene. The faunal remains are attributed to the *Ailuropoda-Stegodon* fauna complex of Southeast Asia. Multiple dating methods have been applied to the site, and Bayesian analysis of 48 determinations established a geological and archaeological record spanning approximately 300 ka for Yumidong Cave [24].

Jinsitai Cave, located at the eastern end of the mid-latitude, semi-arid Eurasian belt, on the China-Mongolia border, is a rare Palaeolithic cave site with stratified sequence in northern China. The granite cave covers an area of nearly 120 m^2^. Initial excavations in 2000–2001 depleted the deposit extensively, and subsequent excavations focused on the limited remaining sediment [24]. Around 5000 lithic artefacts, 3000 faunal remains and three hearths were discovered at the site, in nine stratigraphic layers (electronic supplementary material, appendix and figure S2). The upper layers contained a Late Upper Palaeolithic assemblage of microblades and bifacially thinned points, alongside the traditional core-and-flake (small flakes) industry which is typical in contemporaneous sites in northern China. The lower layers were dominated by core-and-flake industry, while some distinctive Levallois flakes were described as Mousterian-like artefacts [25,26]. Some researchers regard the presence of this Mousterian-like industry at Jinsitai as evidence of a population dispersal or technological diffusion from the west. The lithic industry from the Mongolian site Tsagaan Agui was recently compared with the Jinsitai Levallois Mousterian [27] but more comparative techno-typological work needs to be done. The Jinsitai fauna is attributed to the *Mammuthus–Coelodonta* faunal complex, although no mammoths are included in the assemblage. Radiocarbon dating on bone collagen suggests human occupation of the cave from around 47–44 ka BP until the Holocene [26].

### (b) Materials

For Yumidong Cave, we randomly selected 121 non-diagnostic bone fragments (no teeth or antler) from layer 2 to layer 9, all of which were excavated in 2013. We limited our sampling to bones from the uppermost Middle Pleistocene and Late Pleistocene layers, due to concerns of collagen preservation. The average size of the sampled bone fragments was approximately 4 cm.

For Jinsitai Cave, we analysed all 745 unidentifiable bones from the site. They were excavated during the 2000–2001 fieldwork but lack exact contextual information. This is because while all bones were collected and grouped during excavation by layers, after the zooarchaeological analysis, fragments lacking morphological characteristics from every layer were mixed together. We used this mixed ‘unidentified’ assemblage for our ZooMS work. During sampling, we noted the presence of glue on the bones from Jinsitai, verified as polyvinyl acetate applied to the bones shortly after excavation. The glue has aged, cracked and concealed possible modifications on the bone surface. The analysed specimens, most of which were long shafts, varied in size, and we recorded their weight before sampling.

### (c) Sampling and data generating

Each bone was subsampled using a circular diamond saw blade. To eliminate surface contaminants such as glue and sediment, a small area of the bone was sandblasted before removing a chip of approximately 20 mg for ZooMS analysis, or approximately 600 mg for radiocarbon dating.

We used the ZooMS acid-insoluble protocol [22,28] for 866 samples. Seven bones from Jinsitai were submitted to the Oxford Radiocarbon Accelerator Unit and were dated using routine ultrafiltration methodologies [29]. More details can be found in the electronic supplementary material, appendix.

### (d) Data processing

The calculation of glutamine deamidation is based on Wilson *et al*. [30]. The amino acid glutamine (Q) in collagen peptides may undergo *post-mortem* deamidation, resulting in a mass shift of 0.984 Da. COL1ɑ1 508–519 (GVQGPPGPAGPR) (marker P1 or cet1 from previous research [31]) contains a single glutamine site identified at *m/z* 1105.5 (non-deamidated) and *m/z* 1106.5 (deamidated). Theoretically, deamidation values range from 0 to 1. A value of 1 indicates no or negligible deamidation in COL1*α*1 508–519 peptides, while 0 indicates nearly complete deamidation. Values greater than 1 may also be observed due to baseline noise, which can distort the relative intensity.

MALDI-TOF spectra were converted from t2d files to mzXML files using T2D converter [32] and processed using the mMass 5.5.0 [33]. Previously published COL1 peptide markers were used for ZooMS-based taxonomic identifications [15,18,28,34–36]. The raw radiocarbon data were calibrated to calendar years using OxCal v. 4.4.4 [37] and the IntCal20 calibration curve [38]. Statistical analysis and visualization were conducted in R [39] with the ggplot2 package [40].

## 3. Results and discussion

### (a) Deamidation

To investigate the influence of local environmental conditions and age on bone collagen preservation and overall ZooMS performance, we analysed the proteomic profiles using glutamine deamidation observed in the peptide COL1ɑ1 508–519. This peptide sequence is conserved across mammalian species and has been used in previous studies as a proxy for the relative ‘thermal age’ of samples or to detect intrusive bones of different ages in an *in situ* deposit [41,42].

**Figure 2.**
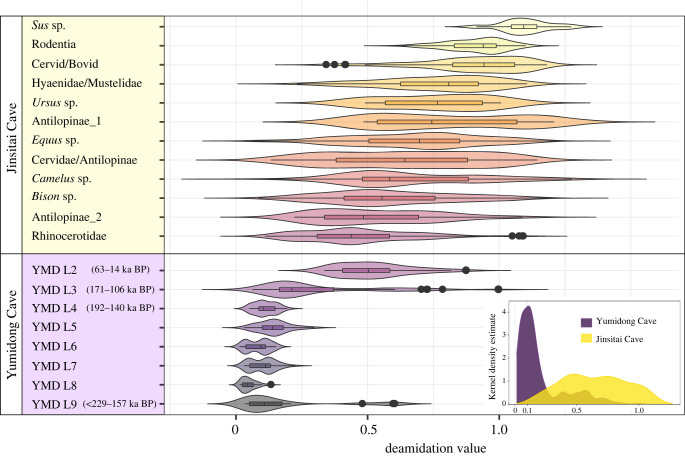
Visualization of deamidation levels at Yumidong and Jinsitai. Insert panel: kernel density estimate of deamidation value on ZooMS identifiable mammals. Main figure: violin plot on deamidation levels for Jinsitai (in yellow, around 47–44 ka BP to the Holocene) grouped by ZooMS-identified taxa, and Yumidong (in purple, Middle and Late Pleistocene) grouped by archaeological layers. The plotted data for Jinsitai includes *Sus* sp., *n* = 4; Rodentia, *n* = 28; Cervid/Bovid, *n* = 47; Hyaenidae/Mustelidae, *n* = 40; *Ursus* sp., *n* = 4; Antilopinae_1, *n* = 9; *Equus* sp., *n* = 189; Cervidae/Antilopinae, *n* = 53; *Camelus* sp., *n* = 31; *Bison* sp., *n* = 125; Antilopinae_2, *n* = 19; Rhinocerotidae, *n* = 121, shown in descending order on the basis of their median deamidation values. The Yumidong dataset includes ZooMS identifiable specimens from layer 2, *n* = 13; layer 3, *n* = 16; layer 4, *n* = 9; layer 5, *n* = 10; layer 6, *n* = 15; layer 7, *n* = 15; layer 8, *n* = 14; layer 9, *n* = 8, totally 100 (data in appendix, electronic supplementary material, table S1). Chronological data for Yumidong from [24].

Deamidation values for both sites deviated from a normal distribution (electronic supplementary material, appendix and figure S3–S5). Therefore, we used non-parametric Kernel density estimation to assess the overall deamidation patterns (figure 2, insert). The two sites had distinct deamidation patterns. The average deamidation value at Yumidong was lower and less variable than Jinsitai. Despite their wide variation, the median deamidation value for the Jinsitai dataset was 0.62, whereas the value for Yumidong was significantly lower at approximately 0.15. This suggests that, in general, bones from Jinsitai were less deamidated, which agrees well with the higher ZooMS identification rate (see next section), as well as the younger overall age of Jinsitai. Based on the published chronology for each site, the deepest layer at Jinsitai post-dates 50 ka BP. Therefore, the entire Jinsitai deposit corresponds only to the upper part of layer 2 (63–14 ka BP) of Yumidong (electronic supplementary material, figures S1 and S2) [24,26]. To explore further whether the deamidation patterns in both sites were linked to time, we plotted the deamidation values against the ZooMS taxa of Jinsitai (labels in yellow) and the stratigraphic layers of Yumidong (labels in purple) on the same figure (figure 2). While most taxa in Jinsitai presented broad and overlapping ranges of deamidation, an indirect time-related pattern can be observed when comparing the deamidation values of *Sus* sp. (pig/wild boar) and Rhinocerotidae (woolly rhinoceros), whose deamidation ranges hardly overlap. The two taxa are thought to be separated temporally at the site. Zooarchaeological study of the Jinsitai fauna reported pigs/wild boars (*n* = 2) exclusively in layer 2, whereas woolly rhinoceros (*n* = 123) were only found in layers 3–8 (data in electronic supplementary material, appendix, table S2) [43]. Layer 2 corresponds to the Holocene, as evidenced by pottery sherds found there, while woolly rhinoceros pre-date the Holocene and are believed to have gone extinct in East Asia around the Allerød oscillation approximately 13 ka [25,44]. The outlier JST 285 (triplicate) in the Rhinocerotidae group in figure 2 suggests a well-preserved specimen, possibly of a younger age. In Yumidong, most of the deamidation variation occurred in the two upper layers, while deamidation levels in layers 4–9 were close to 0. There was one exception, YMD 113 (Cervidae/Antilopinae) from layer 9, which exhibited a deamidation value of around 0.5. This outlier might indicate either an intrusion or extraordinarily well-preserved collagen.

The wide range of deamidation values for the glutamine of COL1ɑ1 508–519 in bones from Jinsitai and in layers 2 and 3 of Yumidong suggests that deamidation is still ongoing in these deposits. By contrast, bones from layers 4 to 9 in Yumidong are fully deamidated. This agrees with the data that these layers are considerably older, probably pre-dating 140 ka.

While deamidation values cannot be used directly as an indicator of age, in some cases they can serve as a relative age indicator for chronologically separated fauna groups within a single site. However, it is important to note that the deamidation process is influenced by both diagenetic and laboratory-induced factors [45]. Therefore, we ought to stress that our findings are specific to the sampled deposits. The results of this study support previous research suggesting that the deamidation may be considered an indicator of collagen preservation and a thermal age proxy among different fossil assemblages. However, achieving chronological resolution in absolute terms is extremely challenging—if not impossible [41,42,46,47].

### (b) ZooMS taxonomic results and comparison with zooarchaeological data

Despite the antiquity and location of Yumidong and Jinsitai caves in the subtropical and the temperate zones of East Asia, respectively, the ZooMS-based identification rates were unexpectedly high. Out of the 745 bones analysed from Jinsitai Cave, 90% had enough collagen for assignment to the order or genus level. The success rate for Yumidong Cave is 83%. To assess if the new data fit within the overall zooarchaeological record for each site, we compared the ZooMS-based identifications with the morphological identifications.

In Yumidong Cave, 21 of the 121 analysed samples failed to yield enough collagen. No significant correlation was observed between stratigraphic depth and success of ZooMS identification (electronic supplementary material, appendix and figure S6), possibly due to the limited number of samples included in this study.

A total of 1530 bones were recovered from the first 15 layers of Yumidong, with the majority coming from layers 2, 5, 10 and 11. About one-third of the faunal assemblage (480 specimens) was identified morphologically, revealing a high diversity of taxa (approx. 40) which included *Stegodon* sp.*, Cervus* sp*., Muntiacus muntjac* (southern red muntjac)*,* Caprinae*, Bubalus* sp. or *Bos* sp.*, Sus scrofa* (wild boar)*, Equus* sp. (horse)*, Stephanorhinus* sp. (two-horned rhinoceros), *Megatapirus augustus* (giant tapir), *Ursus thibetanus* (Asian black bear), *Ailuropoda melanoleuca* (giant panda), a few carnivore taxa and microfauna [48]. Based on the abundance of cervids (31%) and stegodons (23%), the Yumidong fauna belongs to the *Stegodon–Ailuropoda* faunal complex, a typical fauna complex of large-bodied mammals widely distributed from East Asia to mainland Southeast Asia during the Late Pleistocene [49–51].

Prior to comparing morphological versus ZooMS datasets, we removed 12 bones of microfauna taxa from the morphological dataset, resulting in a total of 468 morphologically identified mammals [48] (electronic supplementary material, appendix and table S2). We included rodents in our comparison because they account for about 17% of the morphological dataset from Yumidong, contributing to half of the species diversity at the site.

The Yumidong ZooMS results reveal a reduced diversity of mammals compared with the morphological data (electronic supplementary material, appendix and table S2). This may be explained by the fact that (i) the ZooMS dataset (*n* = 100) is nearly five times smaller than the morphologically identified one (*n* = 468); (ii) the ZooMS-analysed bones have an average size of 4 cm, thus most microfauna would have been excluded during sampling; and (iii) the low resolution in separating cervids and bovids using ZooMS could mask the overall taxonomic diversity.

**Figure 3.**
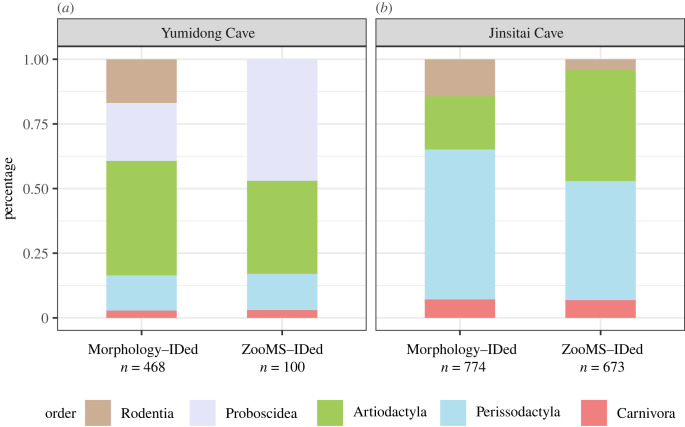
ZooMS results compared with zooarchaeological data for (*a*) Yumidong and (*b*) Jinsitai caves. For each site, the bar plot indicates the percentage of identified specimens of mammals based on morphology and on ZooMS. Further details are provided in the electronic supplementary material, appendix and tables S2–S4.

In order to compare the two datasets, we classified the morphologically identified mammals and the ZooMS-identified mammals into five orders (Rodentia, Proboscidea, Artiodactyla, Perissodactyla and Carnivora) (figure 3*a*).

The abundance of carnivores (e.g. hyenas) in caves is often used to determine whether hominins or carnivores were the driving force for the accumulation of an assemblage [52–54]. The percentage of Carnivora, around 3%, is similar in both datasets of Yumidong, falling below the 20% threshold required for designating a fauna assemblage as carnivore accumulation. This confirms published work on bone and lithic artefacts analysis that highlights the dominant role of hominins in the formation of the site [55,56].

The Perissodactyla group includes extinct regional species, such as two-horned rhinoceros, giant tapirs and very few horses. Rhinos and tapirs share all diagnostic peptide markers [57], leading to a combined Ceratomorpha category in our ZooMS dataset. Interestingly, the percentage of order Perissodactyla is consistent in both ZooMS and morphological datasets (14%) (figure 3*a*).

The Proboscidea group is the most abundant in our ZooMS results. Stegodon, the typical species in the *Stegodon–Ailuropoda* faunal complex in Southern China, represents 22% of the morphological assemblage at Yumidong Cave. Stegodon remains, primarily consisting of cranial and foot elements [58] have been found in all layers at Yumidong, and over 85% were neonate and juvenile individuals. While our current ZooMS reference library lacks stegodon, we identified 47 bones whose spectra matched the Elephantidae ZooMS fingerprint [35]. Since *Elephas* coexisted with extinct stegodons in southern China throughout the Pleistocene, the ZooMS-identified proboscideans from Yumidong are assigned to Elephantoidae, a group that includes both Stegodontidae and Elephantidae. Elephantoidae represents 47% of the ZooMS assemblage, making it the most abundant taxon in the Yumiding ZooMS dataset.

The zooarchaeological studies [48,56] suggest diverse strategies for the exploitation of large animals. The inhabitants of Yumidong scavenged or hunted stegodons but only transferred the skulls and limbs of neonate and juvenile individuals back to the cave. Two stegodon tusks from layers 2 and 5 were modified for the production of tools. The remains of two-horned rhinoceros show a bias towards older individuals and less preference on transporting body elements to the site.

Artiodactyla (mainly cervids and bovids) is the largest group in the morphological dataset but ranks second after Elephantoidae in the ZooMS results. Bovids, *Bos* or *Bubalus*, account for 13% in the morphological dataset but 31% in the ZooMS data. ZooMS can separate *Bos* from *Bubalus*, which is challenging morphologically. However, ZooMS cannot reach genus-level identification for cervids (including *Cervus* sp. and southern red muntjac at the site). The fragmented ZooMS assemblage shows a larger percentage of *Bos* and *Bubalus*, while the proportion of cervid specimens decreases significantly (29% versus 4%). This disparity may be due to body size, with large animals better represented in the ZooMS assemblage. Two cervid antlers were modified into tools [56].

Despite the limited and exploratory nature of the application of ZooMS at Yumidong, our analysis provides a new perspective on the highly fragmented bones from the site. It complements the morphological identifications that dominantly rely on teeth. The least abundant groups (Carnivora and Perissodactyla) in the morphological dataset (3% and 14%) align with the ZooMS-based dataset, while the more dominant orders (Rodentia, Artiodactyla and Perissodactyla) show differences in the ZooMS dataset that may be the result of body-size effects.

In Jinsitai, out of 745 samples analysed using ZooMS, 68 had no collagen. The remaining 677 samples were successfully analysed, with one identified as Aves (bird) and 673 as mammals (electronic supplementary material, appendix and table S4). Three bones were assigned to an 'unknown' category due to unmatched peptide masses.

While all analysed bones lacked diagnostic morphological features, not all of them were small in size. A slight correlation (Cohen's *d* = 0.31) was found between bone weights and ZooMS success rate, with rates of 93% for the greater than 10 g group, 91% for the 3–10 g group and 88% for the less than 3 g group, resulting in an overall identification rate of 90% (electronic supplementary material, appendix and figure S7).

The main excavation of Jinsitai Cave yielded 2372 bones from layers 2 to 8. In total, 778 (33%) specimens were identified morphologically to genus or species level. More than half of the morphologically identified specimens (51%) come from layer 4, while layers 2, 3 and 5 each yielded 11–13%. In addition to four bird bones, 15 mammalian taxa were identified at the site, including *Myospalax aspalax* (zokor), *Marmota bobak* (bobak marmot), *Cervus elaphus* (red deer), *Procapra przewalskii*, *Pachygazella* sp., *Spirocerus* sp., *Bison* sp. (bison), *Equus ferus przewalskii* (Przewalski horse), *Equus hemionus* (Asiatic wild ass), *Sus scrofa* (pig/wild boar), *Coelodonta antiquitatis* (woolly rhinoceros), *Ursus spelaeus* (cave bear), *Crocuta crocuta ultima* (hyena), *Canis lupus* (wolf) and *Gulo* sp. (wolverine). Minimum numbers of individuals (MNIs) were estimated for these taxa (electronic supplementary material, appendix and table S2) [43].

The Jinsitai fauna is attributed to the *Mammuthus–Coelodonta* faunal complex despite the absence of mammoth. The site is located in a relatively open landscape compared with eastern regions where mammoths have been recorded [59,60]. Although no comparable cave site exists in the region, similar taxa, with the exception of cave bear and bobak marmot, were found at the open-air site of Salawusu in Inner Mongolia [61] (figure 1). Sediment pollen analysis suggested a shift from a taiga-steppe to a less-cold steppe ecosystem during the human occupation of Jinsitai Cave.

To compare morphological and ZooMS-identified taxa, the 774 morphologically identified mammals [43] were categorized into four orders (Rodentia, Artiodactyla, Perissodactyla and Carnivora), and they were compared with the 673 ZooMS-identified mammals, also grouped into four groups (figure 3*b*).

The Carnivora category in the ZooMS and the morphological datasets both represent approximately 7% of the assemblage at Jinsitai, mainly made by cave bears and hyenas. Among the morphologically identified specimens, around 160 bones showed traces of burning, over 140 had cut-marks, and less than 40 had signs of carnivore gnawing [43]. Since only one mustelid was identified morphologically, we hypothesize that the indistinguishable taxon Hyaenidae/Mustelidae in ZooMS mostly contains hyenas.

In the morphological dataset, Rodentia accounted for about 14% of the Jinsitai assemblage, represented by bobak marmots (*n* = 108) and zokors (*n* = 3). However, in the ZooMS assemblage, only 4% (*n* = 28) of the bones were assigned to rodents, and they were almost exclusively found in the smallest weight group (less than 3 g). Although bobak marmot and zokor were not present in the ZooMS reference library, the Jinsitai rodents showed closest match to the alpine marmot (CDS: XP_015350976.1). The lower number of rodents in the ZooMS dataset suggests that rodents were not severely fragmented. The deamidation level of rodents indicates a relatively late appearance at Jinsitai, consistent with previous zooarchaeological studies on bobak marmots, which were limited to layers 2 to 4 at Jinsitai and may have been the result of burrowing activity [62]. Bobak marmot is absent at Salawusu, in the same region [43,61].

Artiodactyla is the most diverse group in the Jinsitai faunal assemblage and includes pigs/wild boars, red deer, four bovids (*Procapra przewalskii*, *Pachygazella* sp., *Spirocerus* sp., *Bison*. sp.) [43] and the newly identified *Camelus* sp. (camel) (see text below). Two pig/wild boar remains were morphologically identified both in layer 2, and their presence was confirmed by ZooMS, albeit very infrequent (*n* = 4). Bison accounted for 9% (*n* = 71) of the morphological dataset, but this value doubled in the ZooMS data (18%, *n* = 121), indicating a potentially higher fragmentation level for this taxon. Although morphological and ZooMS analyses could not determine the Jinsitai bison remains to species level, it has been suggested that all Late Pleistocene bison remains in the northeast China plain should be identified as *Bison priscus* (steppe bison) due to the lack of reliably identified alternatives [63]. Red deer is the only morphologically identified cervid at Jinsitai (*n* = 25), with various axial and appendicular elements, as well as four antler fragments. By contrast, the remaining three local Antilopinae taxa, *Procapra przewalskii*, *Pachygazella* sp. (extinct) and *Spirocerus* sp. (extinct), were exclusively identified by horn fragments (*n* = 17, 43 and 1, respectively), which are their most distinctive parts. Almost half of the identified horns had cut-marks at the roots, resulting from the removal activity on crania [43]. Using ZooMS, we were not able to identify any cervid or Antilopinae bones to the genus level at Jinsitai, due to their phylogenetic closeness and absence in the ZooMS reference library. Instead, 129 specimens were grouped into five ‘ZooMS taxa’ based on distinct marker combinations (for more details see the electronic supplementary material, appendix). Of these, the ‘Cervid/Bovid’ group (*n* = 47) represented the most generic assignment due to the lack of one or two ZooMS markers. The remaining four groups (*n* = 82) each represented a combination of seven markers, suggesting the presence of at least four ZooMS unidentified species at Jinsitai. The ambivalent classification of cervids hinders further discussion on the exploitation of cervids or antelopes at Jinsitai.

The order Perissodactyla includes two *Equus* species (Przewalski horse and Asiatic wild ass) and woolly rhinoceros at Jinsitai. Woolly rhinoceros were equally represented in the morphological and ZooMS datasets (16% versus 18%). Based on morphological identification [43], the two *Equus* species were the most abundant taxa, accounting for 42% of the Jinsitai fauna, with 220 Przewalski horse specimens representing 12 individuals and 106 Asiatic wild ass specimens representing 13 individuals. Over 90% of identified elements were teeth and distal extremities, suggesting a possible preference by hominins for transporting skull and lower limbs to the site. Evidence from other sites in adjacent regions [64] shows that Przewalski horse/Asiatic wild ass was a substantial food resource for hominins. Using the ZooMS trypsin-digestion protocol, the Przewalski horse and Asiatic wild ass were indistinguishable and were identified as a combined *Equus* taxon. *Equus* sp. accounted for 28% of the ZooMS assemblage, much less than the morphological dataset. The over-representation of *Equus* in the morphological identifications may be attributed to the distinct morphology of horse teeth [65,66]. At Jinsitai, 67% of the morphologically identified *Equus* remains were teeth (*n* = 219). Interestingly, none of these teeth was from calves or young adults (less than 4 years) [43]; instead, the horse composition suggests a long-term exploitation of prime and old adults at Jinsitai.

The comparison between Jinsitai morphological and ZooMS datasets reveals specific differences. For example, cervids and bovids are more abundant in the ZooMS data, while rodents and *Equus* are less frequently found. The two assemblages represent the entire fauna collection from the main excavations of the site. Although the ZooMS data from Jinsitai presented here lack stratigraphic context, the deamidation analysis detected a few taxa (pig/wild boar and rodents) of which the presence at the site was relatively short and recent. Furthermore, four different ZooMS marker combinations were identified on cervids/bovids, representing at least four species. To achieve a more detailed taxonomic resolution, expanding the ZooMS reference with bovid species found in East Asia is necessary to clarify the new marker combinations [36].

### (c) Camels in Jinsitai Cave

An unexpected discovery was the identification of camel remains in the faunal assemblage of Jinsitai. Thirty-one (*n* = 31) camel bone fragments were discovered using ZooMS (spectra in electronic supplementary material, appendix and figure S8), comprising 5% of the ZooMS-identified dataset. While ZooMS can identify two extant species in the genus *Camelus* at the species level (*C. bactrianus* and *C. dromedarius*) [15], the extinct *C. knoblochi* is not included in the current reference library. It is likely that the *C. knoblochi* shares most, if not all, ZooMS markers with the *C. ferus* (wild bactrian camel). The extinct ‘giant’ camel *C. knoblochi* was part of the *Mammuthus–Coelodonta* faunal complex that inhabited Asia for tens of thousands of years, although the exact date of its extinction remains uncertain [67].

Traditionally, camels are not considered a targeted species for Eurasian Palaeolithic hunter groups, and their remains are rarely found at cave sites. Camel skeletal remains preserve more diagnostic features than other megafauna species, hence their morphological identification should be, in principle, easy to achieve. Their absence, therefore, may be either because their feral predecessors were not numerous in the landscape thus rarely targeted, or, when hunted, transportation of body parts between killing sites and camping sites was limited. Following ZooMS identification, the 31 camel bones were morphologically examined; none preserved diagnostic features and all were heavily fractured. Seventeen fragments were from long bones, three from flat bones and four from irregular bones (electronic supplementary material, appendix and table S4). Like most taxa in the Jinsitai ZooMS assemblage, the size of camel fragments varied considerably, and were equally identified in the less than 3 g and greater than 10 g groups. Two camel long bone fragments showed possible traces of burning, probably due to heating to a low temperature since bone collagen was still present. The highly fragmentary nature of the camel bones discovered at Jinsitai may suggest that the camel bones underwent extensive level of modification and damage pre- and post-deposition. It is possible that while humans exploited camel remains, they only transferred specific body parts to the cave. This could explain the absence of more diagnostic bones (teeth, crania and vertebrae).

**Figure 4.**
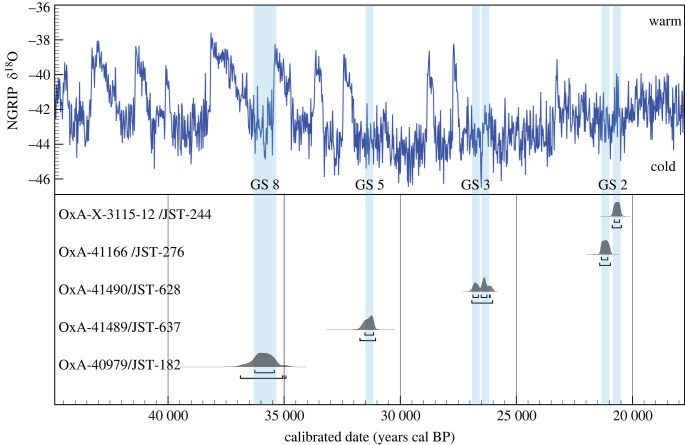
Calibrated probability functions of the radiocarbon ages for five camel bones from Jinsitai (data in electronic supplementary material, appendix, table S5). The raw ages were calibrated using OxCal v. 4.4.4 [37] and the IntCal20 terrestrial curve [38]. The Greenland oxygen isotope record (NGRIP) is shown on top of the graph. The blue shaded lines represent the 68.2% probability ranges of the calibrated radiocarbon ages and appear to correspond well to the cold Greenland stadials [69].

To establish the absolute timing of camel presence at Jinsitai, we radiocarbon dated seven ZooMS-identified camel bones, all of which were dense and/or large fragments. Of these, five produced enough collagen for dating. The results indicate that the five dated specimens represent at least four individuals (electronic supplementary material, appendix, table S5 and figure S9). OxA-X-3115-12 (JST 244) was produced on a low collagen bone and we cannot rule out that this is a minimum age. Notwithstanding, camel bones were deposited at Jinsitai during distinct periods, at 20.5 ka cal BP, at 26 ka cal BP, at 31 ka cal BP and at approximately 36 ka cal BP (ka cal BP = calendar thousand years before present). When plotted against the Greenland oxygen isotope record (NGRIP) [68] these new dates fall in cold conditions of the marine isotope stages (MIS) 3 and 2, particularly Greenland stadials 2.1, 3, 5 and 8 [69] (figure 4). The chronometric data we report here clearly indicates that camel presence at Jinsitai was not ephemeral or a one-off encounter. Instead, it suggests targeted and repeated exploitation of this animal for at least 17 millennia.

The deamidation levels of the dated camel bones were also examined. Four of the five specimens showed deamidation values ranging from 0.4 to 0.6. The exception was JST 628 (approx. 26 ka cal BP), with an average deamidation value of 0.95, indicating a nearly non-deamidated profile. This specimen was not the oldest among the directly dated bones. The inconsistency between radiocarbon age and deamidation level cautions against using deamidation as a molecular ‘clock’.

**Figure 5.**
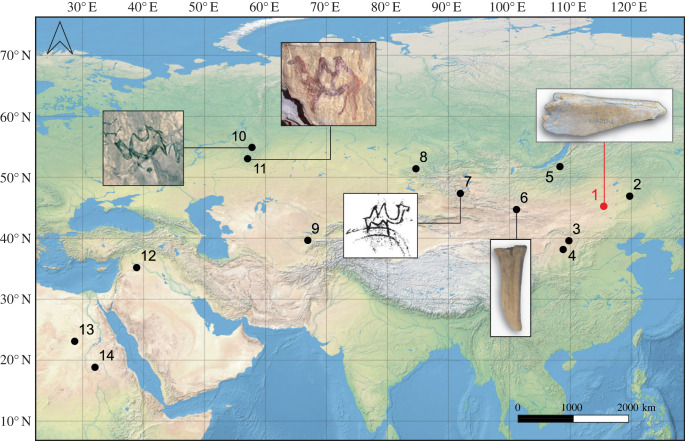
Camel bone remains and camel rock art depictions from Palaeolithic localities. 1, Jinsitai (China); 2, Otson Tsokhio (Mongolia); 3, Wulanmulun (China); 4, Salawusu (China); 5, Kamenka A (Russia); 6, Tsagaan Agui Cave (Mongolia); 7, Khoid Tsenkheriin Agui Cave (Mongolia); 8, Denisova Cave (Russia); 9, Samarkandskaya (Uzbekistan); 10, Ignatievskaya Cave (Russia); 11, Kapova Cave (Russia); 12, Nadaouiyeh Aïn Askar (Syria); 13, Bir Tarfawi (Egypt); 14, HP766 Wadi Umm Rahau (Sudan). Images of rock art provided by V. Shirokov; redrawing of camel rock art from Khoid Tsenkheriin Agui Cave and camel metacarpal from Tsagaan Agui, both modified after [70]. Base map from https://www.naturalearthdata.com/.

In recent years, camel remains have been identified and reported at various Palaeolithic localities in Eurasia and Africa (figure 5). In Western Asia and North Africa, most instances appear to belong to the *Camelus thomasi* (wild dromedary) based on findings from mostly open-air sites in Egypt [71], Sudan [72] and Syria [73], dating to the Middle and Late Pleistocene. In Siberia, camel aDNA has been extracted at Denisova Cave from Middle Palaeolithic sediments dating to 140–120 ka BP [74].

Camelid bones have been reported from early Upper Palaeolithic sites in Uzbekistan (Samarkandskaya) [75], Siberia (Kamenka 1) [76] and Mongolia (Otson Tsokhio) [77,78]. These probably belong to the two-humped wild bactrian camel although there is still uncertainty as to its relationship and time of extinction with *Camelus knoblochi* [67]. Remains of *C. knoblochi*, the largest Eurasian two-humped member of the genus *Camelus*, have been recently reported from Tsagaan Agui Cave in southern Mongolia [70]. In China, *C. knoblochi* bones have been previously identified at the palaeontological locality Dabusu, possibly dating to around 20 ka [79], and a few undated specimens were reported from two Palaeolithic sites, Wulanmulun [80] and Salawusu [81] in Inner Mongolia.

Interestingly, rare instances of Northern Asian parietal cave art found in the southern Urals in Russia (Kapova Cave or Shulgan-Tash, and Ignatievskaya Cave) and Mongolia (Khoid Tsenkheriin Agui Cave), depict two-humped camels among other taxa [82–84] (figure 5). The Urals sites are estimated to date to approximately 20–15 ka BP, while Khoid Tsenkher Cave is believed to pre-date the Last Glacial Maximum. An interesting scene of camel hunting engraved on a mammoth tusk found near River Tom in West Siberia has been dated to minimum 13 ka BP [85].

Among these sporadic occurrences of camel bones and camel depictions, Jinsitai has the most numerous and well-dated camel remains so far. Camel presence there spanned at least 17 millennia. While it is not possible to specify which hominin species targeted camels at each site, it seems that both archaic (Asian Neanderthals and Denisovans perhaps, e.g. at Denisova Cave), as well as early modern humans, were exploiting this taxon. As hunter–gatherer populations expanded across North and Central Asia, they encountered camelids among the diverse megafauna. The extinction of the giant wild camel, *Camelus knoblochi*, probably occurred around 20 ka BP [79], which aligns well with the age determinations at Jinsitai for JST 244 and JST 276. However, further research at other sites is necessary to establish last appearance dates for the species with any confidence. Ongoing studies on the genetic profile of the camel bones discovered at Jinsitai aim to provide a better understanding of the evolutionary history of wild camels in Asia.

## 4. Conclusion

ZooMS has emerged as a valuable biomolecular tool which complements and enhances the traditional zooarchaeological research, especially when dealing with highly fragmented faunal assemblages. In this study, we conducted the first systematic large-scale application of ZooMS in China, analysing nearly 900 bones from two Palaeolithic sites. The analysis of glutamine deamidation at Jinsitai revealed an ongoing deamidation process with a possible temporal correlation to specific taxa. By contrast, at the much older layers of Yumidong, we observed nearly complete deamidation in bones dating back to 140–106 ka BP or before. We successfully extracted bone collagen from Yumidong layers dating back as far as 150 ka, in the subtropical zone of southern China. This opens up exciting possibilities for large-scale screening for collagen and other biomolecules in Pleistocene bones from such latitudes.

We identified 31 camel bones at Jinistai Cave, a previously unknown taxon at the site. Five of these bones were radiocarbon dated to between 37 and 20 ka cal BP; their punctuated presence at the site so far falls in stadial conditions of MIS 3 and MIS 2. The presence of camels at Jinsitai during colder periods provides significant insights into the broad spectrum of animal exploitation performed by early groups of, most likely, modern humans as they spread across the vast ranges of northeast Asia. Such findings highlight the advantages of using novel analytical tools, such as ZooMS, to study non-diagnostic bone assemblages.

Our work highlights the need for the broader application of ZooMS and other biomolecular approaches in East Asia. As ZooMS is applied to new regions, further developmental work is necessary. The current ZooMS reference library contains mostly, if not exclusively, North Eurasian taxa which limits meaningful identification and comparison of results. Given the high success rates, we report here, enlarging the reference library with extinct and extant East Asian taxa and the broader application of collagen fingerprinting to more archaeological assemblages promise exciting results for the future.

## Data Availability

All ZooMS spectra files have been deposited in Mendeley data. (‘ZooMS data of Yumidong and Jinsitai caves', Mendeley Data, V1, https://doi.org/10.17632/ssf27rywhh.2) Supplementary material is available online [86].
